# The Clinical Value of Measuring Circulating HPV DNA during Chemo-Radiotherapy in Squamous Cell Carcinoma of the Anus

**DOI:** 10.3390/cancers13102451

**Published:** 2021-05-18

**Authors:** Anna C. Lefèvre, Niels Pallisgaard, Camilla Kronborg, Karen L. Wind, Søren R. P. Krag, Karen-Lise G. Spindler

**Affiliations:** 1 Experimental Clinical Oncology, Aarhus University Hospital, 8200 Aarhus N, Denmark; anlefe@rm.dk (A.C.L.); klw@oncology.au.dk (K.L.W.); 2 Department of Pathology, Zealand University Hospital, 4000 Roskilde, Denmark; nipa@regionsjaelland.dk; 3 Danish Centre for Particle Therapy, 8200 Aarhus N, Denmark; cam.kro@auh.rm.dk; 4 Department of Pathology, Aarhus University Hospital, 8200 Aarhus N, Denmark; soerkrag@rm.dk; 5 Department of Oncology, Aarhus University Hospital, 8200 Aarhus N, Denmark

**Keywords:** anus neoplasms, squamous cell carcinoma, human papillomavirus, circulating tumor DNA

## Abstract

**Simple Summary:**

Anal cancer is treated with high dose chemoradiotherapy; but despite this, a minor patient group experience treatment failure. Anal cancer is strongly related to human papilloma virus (HPV) infection and previous smaller studies have shown that fragments of HPV DNA can be detected in blood samples from anal cancer patients. This study measured HPV DNA in 88 patients including both small- and more advanced tumors and detected six different subtypes of HPV. During treatment with chemoradiotherapy, the level of HPV DNA decreased, and three elimination patterns with clinical relevance were identified. Fast elimination correlated to a low risk of failure, slow elimination correlated to risk of failure in the pelvis, and persistent HPV DNA after treatment correlated to a high risk of later distant failure. The results add new information to the increasing interest in research of HPV DNA in HPV related cancers and holds great clinical potential.

**Abstract:**

Background and purpose: Circulating tumor DNA (ctDNA) is investigated in various cancers. In squamous cell carcinoma of the anus (SCCA) infection with human papilloma virus (HPV) is found in around 90% of cases and here, plasma HPV (pHPV) can be used as ctDNA. Preliminary data have proved the ability to detect pHPV16 and -18 in SCCA. We have developed a highly sensitive method for measurement of six relevant pHPV subtypes, to investigate the elimination pattern of pHPV during chemo-radiotherapy (CRT) for SCCA and its clinical value. Material and methods: Patients treated at Aarhus University Hospital from 2016–2020 were included. P16 status in the primary biopsy was measured and 82% of patients had P16 positive tumor. Blood samples were collected prior to treatment (PT), mid treatment (MT), end of therapy (EOT), and during follow-up (FU). An in-house multiplex digital droplet PCR method measured pHPV subtypes 16, 18, 31, 33, 51, 58. Results: Samples from 88 patients were drawn PT (*n* = 73), MT (*n* = 72), EOT (*n* = 64) and during FU (*n* = 41). Plasma HPV was detectable in 52 patients and PT pHPV levels correlated to tumor stages. Three elimination patterns were observed during CRT with correlation to outcome: fast responders with no local or distant failures (0/12); slow responders with high risk of local failures (4/20), no distant failures; persistent molecular responders with high risk of distant failures (4/13), but no local failures, *p* < 0.01. Conclusion: During CRT, pHPV can divide patients with SCCA into three groups with significantly different risk of failure. The use of pHPV can potentially assist in clinical treatment decision.

## 1. Introduction

Squamous cell carcinoma of the anus (SCCA) is a rare disease with increasing incidence [1,2], and is related to infection with human papilloma virus (HPV) in more than 80% of cases [3,4]. In HPV related squamous cell carcinomas (SCC) the virus is integrated in the tumor genome, causing uncontrolled virus protein transcription, including the E6 and E7 proteins. These proteins accelerate cell division, and consequently the tumor suppressor protein P16 is upregulated in HPV related tumors [5].

Localized SCCA is treated with high-dose chemo-radiotherapy (CRT) leading to an excellent local control of approximately 87 percent [6]. However, CRT implies a high risk of acute and/or late side effects, with potential negative impact on quality of life [7,8,9,10]. Development of distant metastases occurs in approximately 10 percent of the patients after primary treatment and holds a poor prognosis with a median survival of 12–20 months [2,11]. There is an unmet need for tools to identify the minor but important patient group that may benefit from a more intensive primary treatment or adjuvant therapy and secondly to identify patients that could achieve tumor control with a deescalated treatment strategy thereby avoiding unnecessary side effects.

Circulating free DNA (cfDNA) is a mixture of normal cell DNA fragments and tumor DNA fragments (ctDNA) [12] in the blood. The constant release and fast elimination make ctDNA a timely marker for early evaluation and detection of recurrence after primary therapy [13,14,15,16,17]. In HPV related tumors, as most SCCA [3], ctDNA measured as serum HPV or plasma HPV (pHPV), has been detected in blood samples from patients with oropharynx cancer, cervical cancer, and in smaller studies for SCCA [18,19,20,21,22,23,24,25,26]. Published studies have focused on detection of the most common HPV subtypes 16 and 18; however, the family of HPV holds more than 100 subtypes of which a minor group is described in relation to SCCA [4].

Consequently, we have developed a highly sensitive multiplex digital droplet PCR (ddPCR) method detecting the HPV subtypes 16, 18, 31, 33, 51, and 58 in blood samples from a cohort of 88 patients treated with CRT for SCCA.

The aims of this study were to evaluate the prognostic value of pHPV in relation to pre-treatment characteristics, asses the clinical value of pHPV elimination patterns, and to determine the landscape of HPV subtypes detected in pHPV in SCCA.

## 2. Materials and Methods

Patients with newly diagnosed SCCA at Department of Oncology, Aarhus University Hospital, Denmark, were included in the prospective clinical data and biobank collection study “Plan-A”, Ethical Committee no. 1-10-72-79-16, as described previously [9,13].

Blood samples were collected prior to CRT (PT) mid through CRT (MT) (+/−7 fractions) and at end of CRT (EOT) and in the follow-up period one to three years after CRT (FU).

Plasma was centrifuged at 4 °C at 2000 *g* for 10 min within two hours and stored at −80 °C until further analysis, according to the standard operating procedures from Bio- and Genome Bank, Denmark [27].

A 191 base pair (bp) spike-in DNA [28] was added to 4 mL of thawed plasma and DNA purified on a Chemagic 360 robot (PerkinElmer, Waltham, MA, USA) using a 1304 cfDNA purification kit (PerkinElmer). To control sample fragmentation and cfDNA amount an in-house multiplex ddPCR reaction was performed amplifying a 65 bp and 250 bp fragment of the EMC7 gene using a QX200 AutoDG ddPCR system (Bio-Rad, Berkeley, CA, USA). The EMC7 gene on chromosome 15 was chosen as reference for cfDNA measurement since in cancer this gene has not been reported to be mutated, it is located close to the centromere and therefore unlikely to be involved in chromosomal gains or losses, and no EMC7 pseudo-genes has been reported or found when the human genome was searched.

The spike-in fragment and possible white blood cell (WBC) DNA contamination was measured by multiplex ddPCR. The WBC assay measures B-cell rearranged immunoglobulin genes as previously described [28]. All samples showed acceptable low WBC levels with values below 0.20% and only three samples had concentrations above 4% (11.7%, 9.7%, and 7.7% respectively). During method development a pilot study was conducted on 10 samples with detection of HPV subtype 16, resulting in further method development with a pre-amplification step for increased sensitivity. Consequently, pilot measurements were excluded from quantitative analysis, and only positive pilot measures were included in the overall concordance analysis.

Plasma HPV subtype analysis and quantification were performed on 25 µL purified DNA from the samples and pre-amplified by a 12 cycle PCR reaction, using the Q5^®^Taq polymerase (New England Biolabs, Ipswich, MA, USA) and a multiplex primer-mix targeting the E6 or E7 gene of the relevant HPV subtypes 16, 18, 31, 33, 51, and 58, as well as the EMC7 gene. After pre-amplification the Q5^®^Taq polymerase was inactivated 10 min at 99 °C. The pre-amplificated material comprised approximately 4000 times the original DNA amount and was afterwards diluted 50 times. Finally, six multiplex ddPCR reactions targeting the EMC7 gene and one of the six HPV subtypes were performed and measured using the Bio-Rad QX200 AutoDG ddPCR system. For further method details see Tables S1 and S2 and Figure S4. Responsible laboratory collaborators were blinded for patient characteristics.

P16 immunohistochemistry staining was performed according to standard protocols on paraffin-embedded formalin fixed diagnostic tumor biopsies using an automated system (Ventana, Roche, Switzerland) with the mouse monoclonal antibody Clone E6H4. A cut off level of 70% P16 positivity, was used for classification [3,29].

Plasma HPV was reported as percentage of total EMC7-65. Wilcoxon–Mann–Whitney test and Kruskal–Wallis equality-of-populations rank test were used for comparison between pHPV and pre-treatment characteristics. Elimination patterns for pHPV are presented on scatterplots of raw data and required a minimum of two measurements per patient during CRT including an EOT measurement, unless pHPV elimination was detected from PT to MT. The association between response patterns and risk of failure was calculated with Fisher’s exact test. Disease free survival (DFS) (defined as date of inclusion prior to CRT until first coming event: progression or non-response within 6 months after CRT, local- or distant relapse, or death of any cause) and overall survival (OS) (defined as time from inclusion to death of any cause) were presented using the Kaplan–Meier plot. Outcome comparison between patients with PT pHPV level below and above the median pHPV level was analysed using log-rank test and cox regression, presented with hazard ratios (HR). Multivariate analysis was not preformed due to low number of events. *p*-values below 0.05 were considered significant. Statistical analyses were done using STATA/IC16.0 (Stata Corp LP, College Station, YX, USA).

## 3. Results

### 3.1. Patient Characteristics

A total of 88 patients were included. The median follow-up time was 29 months (range 9–53). Pre-treatment characteristics are presented in Table 1. Most patients were female (74%), the median age 62 years (range 26–84) and the majority presented with tumor stage (T) 1–2 tumors (82%). Less frequent was T3-4 (18%), and lymph node (N) positive disease (24%). Most tumors were P16 positive (82%). Patients were treated as previously presented [9,13]. Radiotherapy comprised dose levels of 64Gy/32 fractions (*n* = 58), 60Gy/30 fractions (*n* = 17) and 54-50Gy/27-25 fractions (*n* = 12), one patient received 48Gy/30 fractions. Cisplatin based concomitant chemotherapy was administered to 64% of patients. The overall local control rate was 90%, but four patients (4%) did not respond to CRT and five patients (6%) experienced a local recurrence. Four patients (4%) with complete response to CRT experienced subsequent distant failure. The two years DFS and OS were 85% (95% CI, 75–91) and 98% (95% CI, 89–99.77), respectively.

### 3.2. Plasma HPV Sample Collection

Blood samples were available from 73 patients PT, 72 at MT and from 64 at EOT. Finally, 41 patients had FU samples drawn. Sample distribution is presented in Table 2.

### 3.3. P16 Status and pHPV Subtypes

**Figure 1:**
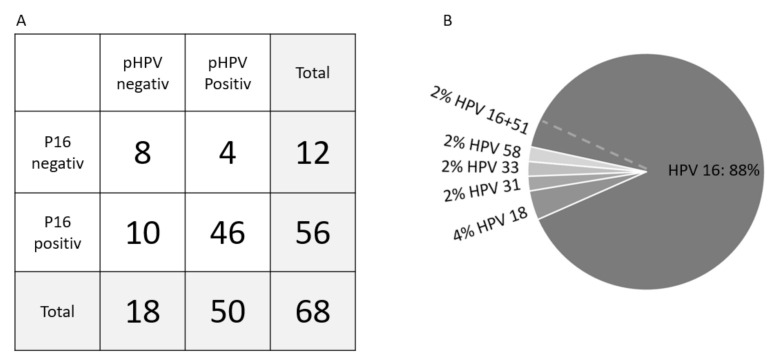
Concordance between tumor P16- and pHPV status (**A**) and pHPV subtype distribution (**B**). (**A**) Concordance between P16 immunohistochemically staining and pHPV status showed a concordance of 79% and a pHPV sensitivity of 82% and specificity of 67%. (**B**) The distribution of HPV subtypes with 90% HPV16 and 4% HPV18. The rare subtypes (31, 33, and 58) contributed with 2% each. One sample was positive for both subtype 16 and 51.

The overall concordance between P16 and pHPV status was 79%, the sensitivity 82% and specificity 67% for the total cohort, as presented in Figure 1A. The sensitivity was 90% in the high-risk group alone (*n* = 25). The most frequent HPV subtype identified in the plasma was HPV16 (90%), Figure 1B. There was no significant difference in pHPV levels and clinical response, DFS or OS according to subtypes.

The mean and median PT pHPV levels in the cohort with pre-amplificated measurements were 9.02% and 0.21%, respectively (range 0.00–180.90). A small group of patients (*n* = 8) had PT samples with ultra-high pHPV levels, some of which exceeded the amount of cfDNA alleles with a median level of 45.00% (range 15.11–180.90), compared to 0.09% (range 0.00–9.82) for the remaining patients (*n* = 55). A scatter plot of the sample distribution is illustrated in Figure S1. There was no significant difference in pre-treatment characteristics or treatment outcome between patients with ultra-high pHPV level and the remaining group. The biological explanation for these observed ultra-high pHPV levels is to be further elucidated.

### 3.4. Circulating HPV Levels and Pre-Treatment Characteristics

**Figure 2:**
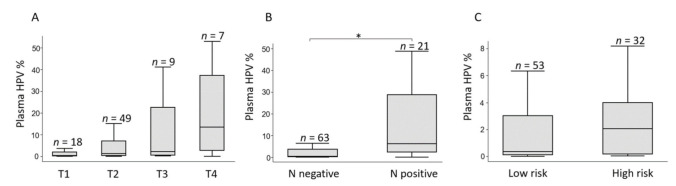
Pre-treatment pHPV levels according to disease stage. Boxplots with median pHPV value in % of total level of circulating free DNA. Lines are ranges. Outliers (*n* = 8) were removed. (**A**) The pHPV level increases non-significantly with increasing tumor (T) stage. (**B**) Comparison of pHPV between lymph node negative (N negative) and lymph node positive (N positive) tumors. (**C**) A higher level of pHPV is detected for T2-T3 and/or N positive tumors (high-risk) compared to T1-T2, N negative tumors (low risk). Statistical significance (*) were calculated using Wilcoxon–Mann–Whitney test.

The pre-treatment characteristics and pHPV levels are shown in Table 1. The median PT pHPV increased with advancing T stage (T1: 0.35%, T2: 1.26%, T3: 2.29%, T4: 13.46%), but the differences did not reach statistical significance, *p* = 0.39 (Figure 2A) and there was a significantly higher pHPV level in patients with lymph node positive compared to lymph node negative disease, 6.09% (95% CI 2.08–34.54) and 0.39% (95% CI 0.18–2.59), respectively, *p* = 0.02 (Figure 2B). A higher level of pHPV was detected for high-risk tumors (T3-T4 and/or N positive), median 3.74% (95% CI 0.37–19.75) compared to low-risk tumors (T1-T2, N negative), median 0.39% (95% CI 0.19–3.05), although not statistically significant, *p* = 0.09 (Figure 2C).

### 3.5. Elimination Patterns of pHPV during CRT

**Figure 3:**
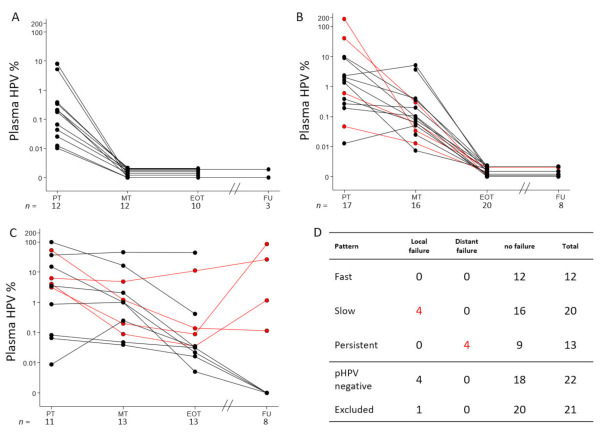
Elimination patterns of pHPV. Patients with measurable pHPV during treatment were divided into three groups. (**A**) 12 patients with a fast pHPV elimination pattern. (**B**) 20 patients with slow pHPV elimination pattern. (**C**) 13 patients with molecular persistent disease. In (**D**), the relation between the pattern and treatment outcome are outlined, including 22 patients with undetectable pHPV and the 21 patients who were excluded due to insufficient repeated pHPV measurements. Highly significant differences were detected between all patterns, Fishers exact *p* < 0.01. Red lines represent patients with treatment failure. *X*-axis show sample collection time: Prior to treatment (PT), Mid treatment (MT), End of treatment (EOT) and at one to three years follow up (FU). *Y*-axis measure the level of plasma HPV in percent of total DNA level. Horizontal lines with a pHPV value of zero are separated to illustrate the progression of each case, and sample size (*n*) is measured below each time point.

Three distinct pHPV elimination patterns were detected. The first group of 12 patients showed a “fast molecular response” (Figure 3A) with pHPV elimination at MT. No patients with this pattern experienced local- or distant treatment failure (0/12).

The second group included 20 patients with a “slow molecular response” (Figure 3B) with detectable pHPV at PT and/or MT. All these patients eliminated pHPV at EOT. Four patients in this group experienced local treatment failure (4/20, 20%), but no distant recurrences were observed (0/20) during the study period.

The third group of 13 patients had “molecular persistent disease” (Figure 3C) with measurable pHPV in the EOT sample. No patients in this group experienced local failure (0/13), but four patients were later diagnosed with distant recurrence (4/13, 31%).

Twenty-two patients did not show detectable pHPV in the PT or pilot sample. Four (4/22, 18%) of these later experienced a local treatment failure.

There was no significant difference between ‘fast responders’, ‘slow responders’, patients with ‘molecular persistent disease’ or patients with pHPV negative status regarding distribution of T- and N classification, *p* = 0.81 and *p* = 0.37 respectively.

At time of MT sample patients had received either 16 +/−7 fractions (*n* = 29) or 15 +/−7 fractions (*n* = 12) or 13 +/−7 fractions (*n* = 4). The median treatment time at MT samples collection was three weeks (15 treatment days (range 10–23)). The Level of the MT pHPV level with relation to number of treatment days is outlined in Figure S2.

Twenty-one patients were excluded from elimination pattern analysis due to lack of repeated measurements. Fisher’s exact test for difference between all patterns and outcome was highly significant with *p* < 0.01 (Figure 3D).

Of note, all patients with distant recurrence after initial treatment response had molecular persistent disease at EOT with median 0.12% (95% CI 0.04–11.17) and experienced a further pHPV increase in the FU sample, 13.96% (95% CI 0.11–86.58), *p* = 0.14.

### 3.6. Prognostic Value of pHPV

**Figure 4:**
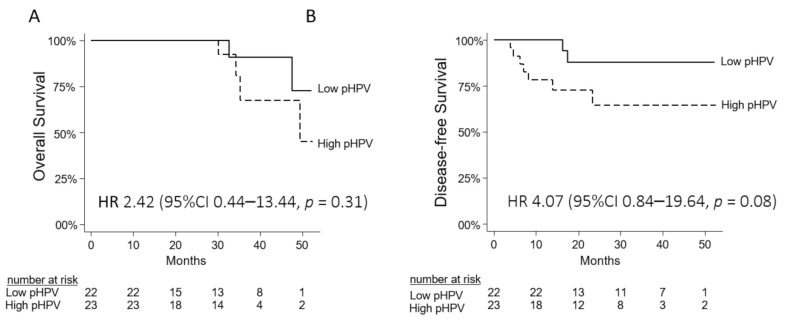
Kaplan–Meier curves for overall survival (**A**) and disease-free survival (**B**) for 45 patients according to high and low pHPV status prior to treatment (cut-off median pHPV of 1.34%).

A difference in OS with a HR of 2.42 (CI 0.44–13.44) *p* = 0.31 was detected when comparing high- versus low median PT pHPV levels. The same was observed for DFS with a HR of 4.07 (CI 0.84–19.64) *p* = 0.08. The number of events was however low. Kaplan–Meier curves are illustrated in Figure 4, and in Figure S3.

## 4. Discussion

This study describes three clinically relevant elimination patterns of pHPV during and after primary CRT for SCCA and investigates the prognostic value of pHPV prior to treatment as well as methodological and biological aspects of pHPV, when using a multiplex ddPCR targeting the most relevant HPV subtypes.

We have developed a multiplex assay to target relevant subtypes for SCCA and demonstrates the presence of the rarer subtypes in pHPV measurement, indicating a clinical relevance of including these in future studies.

Our data shows a high sensitivity (82%) and specificity (67%) of pHPV and P16 status when evaluating both high- and low-stage tumors together. In line with previous studies [23], the sensitivity increased (90%) when evaluating only high stage tumors. The disconcordant cases with P16 negative and pHPV positive results can be explained by relevant biological observations in three cases. One patient was diagnosed with a different HPV associated cancer. Another case harboured the HPV subtype 58, previously described as inversely related to P16 [30]. In the third case there was a 50% P16 positivity, which was below the cut off value of 70%, adopted from HNSCC studies [3,5,31]. P16 staining in SCCA is performed on small diagnostic biopsies potentially hampering a clear conclusion due to P16 heterogeneity in the tumor [32]. There is an unmet need to establish an SCCA specific cut off for P16 positivity. Furthermore, it is necessary to evaluate the relation between P16 and pHPV since P16 staining may not be a perfect surrogate for tumor HPV status [33].

The lack of primary pHPV detection in the P16 positive cases could be attributed to both methodological and/or biological factors. However, several methodological steps were applied to increase the sensitivity of the assay, including pre-amplifications and volume optimization, resulting in a sensitivity ranging from 0.01–0.001%. Contamination of circulating DNA from normal cells can falsely increase the total cfDNA concentration hampering detection of low frequency ctDNA. Test for contamination with WBC was consequently performed on all samples and only a minority of samples were WBC positive, and no samples were excluded from interpretation. Biological factors influencing pHPV detection includes the tumors ability to shed ctDNA which can be affected by both tumor size and the mitotic rate of the tumor [34,35].

Interestingly, a subgroup of samples showed ultra-high pHPV levels. This could be due to multiple HPV genome integrations [36] or as suggested that the integration of the HPV in tumor cells can comprise an episomal component resulting in significantly higher levels of viral load [37,38]. These aspects warrant further investigation but did not influence the conclusion in this study.

Analyses revealed that the pretreatment concentration of pHPV showed a trend towards correlation between stage and level of pHPV, in line with previous observations in other tumor types, and from smaller observations in SCCA [18,23]. Analysis in larger sample sizes are however warranted.

This study provides clinically relevant data with a high sensitivity even for the smallest tumors and describes clinically relevant elimination patterns. Few recent studies have previously presented detection of pHPV in stage I tumors across diagnosis [19,20,24,25,26], and no previous studies have described elimination patterns like the data presented here. These patterns are highly relevant as they correlate to clinically different outcomes: fast molecular responders with low risk of recurrence, slow molecular responders with an increased risk of local recurrences, and patients with persistent molecular disease with a significant risk of distant recurrence. Two previous studies on SCCA (*n* = 18 and *n* = 21) has, in line with this study, described that lack of complete elimination at EOT is associated with a poorer prognosis [23,25]. Although this is the largest study to date, definitive conclusions are limited by the sample size and number of events, but it shows a strong signal which calls for further validation. Patients with fast pHPV elimination and low risk of recurrence could be considered for adaption of CRT with a less intensified treatment in the last weeks of CRT, and thereby decreased risk of unnecessary side effects. Slow responders could be offered more relevant follow-up regimes or CRT with intensified doses in the last weeks of CRT, adapted to the risk of local recurrences. Finally, patients with persistent molecular disease could be followed with frequent pHPV measurements in the weeks after CRT, until molecular complete response, and patients with stable or increasing levels could be offered adjuvant systemic treatment to decrease the risk of distant treatment failure.

The number of samples collected during follow-up was limited, but we observed single cases with a significant lead-time between pHPV detected molecular recurrence and relapse detected by imaging (), suggesting a strong potential for early detection of local or distant recurrences, as also demonstrated in colorectal cancer [17]. These aspects need validation in larger and more mature sample sizes. If confirmed, post CRT pHPV detection and tailored intensified follow-up are natural focuses for prospective studies, and adjuvant systemic therapy could be investigated in clinical trials for patients with pHPV positive EOT samples.

## 5. Conclusions

With a highly sensitive multiplex ddPCR method, this study demonstrates the clinical potential of pHPV in support of treatment decisions, treatment evaluation, and potential future tailored treatment for SCCA.

## Figures and Tables

**Table 1:** Pre-treatment characteristics.

Patient Characteristics	Total Cohort *n* = 88 (%)	Measurable pHPV *n* = 45 (%)	Median pHPV in % of Total cfDNA (95% CI)	*p*-Value
Age (years)				
Median, range	62, 26–84			
<62	44 (50)	26 (58)	1.10 (0.28–4.15)	
>62	44 (50)	19 (42)	2.05 (0.17–10.91)	0.87
Sex				
Female	65 (74)	36 (80)	1.46 (0.28–4.93)	
Male	23 (26)	9 (20)	1.34 (0.04–3.90)	0.35
Performance status (PS)				
PS = 0	62 (70)	32 (71)	1.85 (0.21–4.00)	
PS > 0	26 (30)	13 (29)	0.86 (0.05–39.20)	0.84
P16 status				
Positive	72 (82)	39 (87)	1.66 (0.27–4.07)	
Negative	13 (15)	4 (9)	0.19 (0.02–53.03)	0.40
Unknown	3 (3)	2 (4)	2.41 (1.34–3.48)	0.80
Stage				0.39 *
T1	19 (22)	9 (20)	0.35 (0.04–3.51)	
T2	53 (60)	28 (62)	1.26 (0.23–4.36)	
T3	9 (10)	4 (9)	2.29 (0.18–41.24)	
T4	7 (8)	4 (9)	13.46 (0.03–53.03)	
N negative	67 (76)	33 (73)	0.39 (0.18–2.59)	
N positive	21 (24)	12 (27)	6.09 (2.08–34.55)	0.02
M negative	87 (99)	44 (98)	1.50 (0.29–3.63)	
M positive	1 (1)	1 (2)	0.08	0.25
Risk				
Low	56 (64)	27 (60)	0.39 (0.19–3.05)	
High	32 (36)	18 (40)	3.74 (0.37–19.75)	0.09

Wilcoxon Mann–Whitney test for significance *p* < 0.05. (*) Kruskal–Wallis equality-of-populations rank test.

**Table 2:** Sample distribution.

Number of Available and Excluded Blood Samples	Number
Total:	*n* = 88
Pre-Treatment (PT) (*n* = 63) + Pilot (*n* = 10)	73
Mid Treatment (MT)	72
End of Treatment (EOT)	64
Follow-up (FU)	41
Plasma HPV (pHPV) detection:	*n* = 88
Detectable pHPV (PT or MT + Pilot)	52
Not measurable pHPV (PT + pilot) *	23
Missing PT and undetectable pHPV in remaining samples	13
P16 status and pHPV subtypes **	*n* = 68
PT (43 pHPV positive, 18 pHPV negative)	61
Pilot pHPV positive	5
MT pHPV positive if missing PT sample	2
Pre-treatment characteristics and survival analysis	*n* = 45
PT pHPV positive	45
Elimination patterns ***	*n* = 67
PT + Pilot positive	65
MT	60
EOT	59

* One patient with undetectable pHPV in PT, MT, and FU samples had detectable pHPV in EOT. ** 20 patients were excluded (13 had missing PT and undetectable pHPV in remaining samples, 2 PT positive had missing P16 immunohistochemical staining, 5 pilot negatives were excluded due to incomplete HPV subtype measurement). *** 21 patients were excluded due to lack of repeated measurements.

## Data Availability

The data presented in this study are available on request from the corresponding author. The data are not publicly available due to legal restrictions.

## Supplementary Materials

The following are available online at https://www.mdpi.com/article/10.3390/cancers13102451/s1, Figure S1: Comparison between the amount of plasma HPV with relation to the level of total circulating free DNA measured by the EMC7-65 gene from 63 patients with pre-amplificated pre-treatment samples available. Figure S2: Plasma HPV changes from the pre-treatment to mid-treatment sampling with relation to number of treatment days. Figure S3: Overall survival and disease-free survival according to pre-treatment plasma HPV (pHPV) levels for patients with detectable and undetectable pHPV prior to treatment. Figure S4: Examples of ddPCR results for each of the six HPV subtypes. Table S1: Primers and probes sequences used in the eight multiplex ddPCR. Table S2: Samples used in the development of the multiplex HPV assays.
