# Supplementary material for: The Clinical Value of Measuring Circulating HPV DNA during Chemo-Radiotherapy in Squamous Cell Carcinoma of the Anus
Source: Cancers (Basel). 2021 May 18;13(10):2451. doi: 10.3390/cancers13102451 (PMC8158133; doi:10.3390/cancers13102451)
Supplement: Supplementary file 1 [file cancers-13-02451-s001.zip › cancers-1192879-supplementary.pdf]

Supplementary Materials:

The Clinical Value of Measuring Circulating HPV DNA During Chemo-Radiotherapy in Squamous Cell Carcinoma of The Anus

Anna C Lefèvre, Niels Pallisgaard, Camilla Kronborg, Karen L Wind, Søren R P Krag and Karen-Lise G Spindler

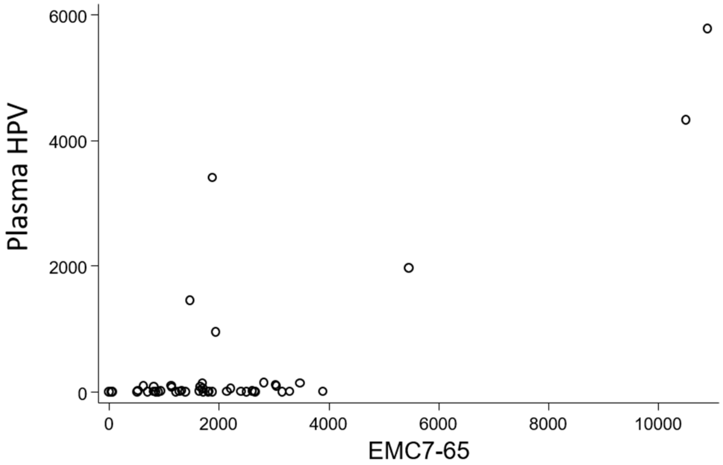

Figure S1. Comparison between the amount of plasma HPV with relation to the level of total circulating free DNA measured by the EMC7-65 gene from 63 patients with pre-amplificated pre-treatment samples available. Most patients (n = 55) had a plasma HPV level below 10% of the total cell free DNA level (EMC7-65), with a median level of 0.09% (range 0.00–9.82%). Eight patients had ultra-high pHPV levels, some of which exceeded the amount of cfDNA alleles with a median level of 45.00% (range 15.11–180.90).

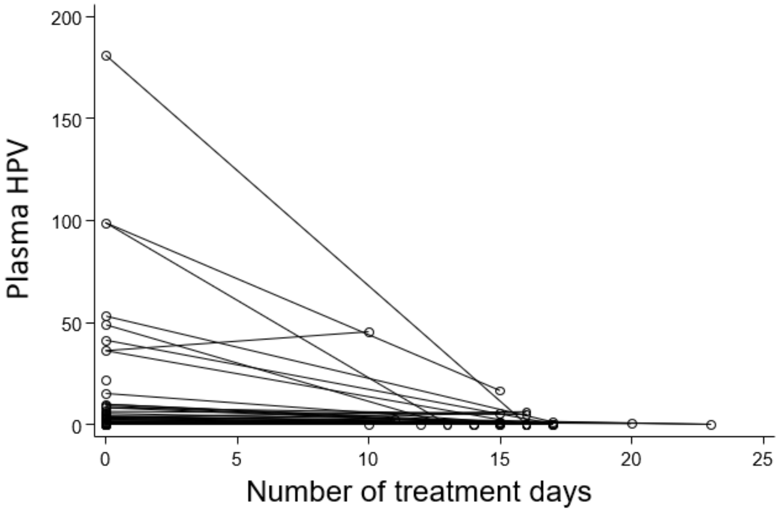

Figure S2. Plasma HPV changes from the pre-treatment to mid-treatment sampling with relation to number of treatment days. Plasma HPV is measured as percent of total EMC7 level.

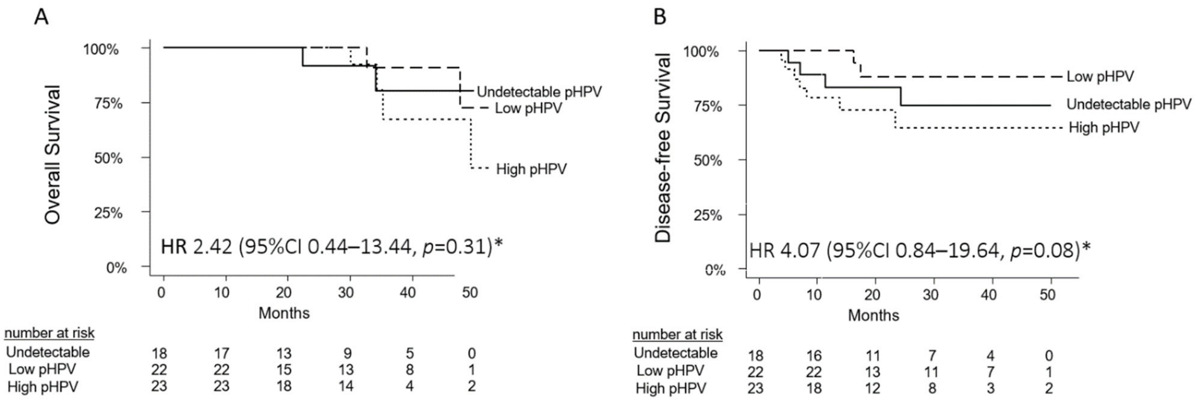

Figure S3. Kaplan-Meier curves for overall survival (**A**) and disease-free survival (**B**) for 45 patients according to high and low pHPV status prior to treatment (cut-off median pHPV of 1.34%) and 18 patients with undetectable pHPV. *Hazard ratios (HR) between patients with high- and low pHPV levels.

Table S1. Primers and probes sequences used in the eight multiplex ddPCR.

ddPCR multiplex primer/probe	Primer name	Sequence
	HPV16 forward	5' CTC AGA GGA GGA GGA TGA A 3'
	HPV16 reverse	5' TGG GCT CTG TCC GGT 3'
**HPV16**	HPV16 probe	5' FAM-ATA GAT GGT CCA GCT GGA CAA GCA-BHQ1 3'
	EMC7 65 forward	5' CTT TCC CCA TGT TGC TTT AT 3'
	EMC7 65 reverse	5' CTG ACA ACC TCT GAT GTT TT 3'
	EMC7 65 probe	5' HEX-CAG AGC AAG ATA TGT GAA TTA CAT CAA-BHQ1 3'
	HPV18 forward	5' GCC AGA ATT GAG CTA GTA GTA 3'
	HPV18 reverse	5' AAA CAG CTG CTG GAA TG 3'
**HPV18**	HPV18 probe	5' FAM-CTC GAA GGT CGT CTG CTG AGC TTT C-BHQ1 3'
	EMC7 65 forward	5' CTT TCC CCA TGT TGC TTT AT 3'
	EMC7 65 reverse	5' CTG ACA ACC TCT GAT GTT TT 3'
	EMC7 65 probe	5' HEX-CAG AGC AAG ATA TGT GAA TTA CAT CAA-BHQ1 3'
	HPV31 forward	5' GAA CTA AGC TCG GCA TT 3'
	HPV31 reverse	5' TTG CAG TAG ACA CAA TTC AA 3'
**HPV31**	HPV31 probe	5' FAM-TCT TAG TTC ATC GTA GGG TAT TTC C-BHQ1 3'
	EMC7 65 forward	5' CTT TCC CCA TGT TGC TTT AT 3'
	EMC7 65 reverse	5' CTG ACA ACC TCT GAT GTT TT 3'
	EMC7 65 probe	5' HEX-CAG AGC AAG ATA TGT GAA TTA CAT CAA-BHQ1 3'
	HPV33 forward	5' AGG TAC TGC ACG ACT AT 3'
	HPV33 reverse	5' GCA CAA ATC ATG CAA TGT TC 3'
**HPV33**	HPV33 probe	5' FAM-TTT CAA GAC ACT GAG GAA AAA CCA C-BHQ1 3'
	EMC7 65 forward	5' CTT TCC CCA TGT TGC TTT AT 3'
	EMC7 65 reverse	5' CTG ACA ACC TCT GAT GTT TT 3'
	EMC7 65 probe	5' HEX-CAG AGC AAG ATA TGT GAA TTA CAT CAA-BHQ1 3'
	HPV51 forward	5' GAT TTA TCG ATA AGG TGT CAT 3'
	HPV51 reverse	5' ACC AAT TTT TGC TTT TCT TCA 3'
**HPV51**	HPV51 probe	5' FAM-AGA TGT CAA AGA CCA CTT GGG CCT-BHQ1 3'
	EMC7 65 forward	5' CTT TCC CCA TGT TGC TTT AT 3'
	EMC7 65 reverse	5' CTG ACA ACC TCT GAT GTT TT 3'
	EMC7 65 probe	5' HEX-CAG AGC AAG ATA TGT GAA TTA CAT CAA-BHQ1 3'
	HPV58 forward	5' GGA GAC ATC TGT GCA TGA A 3'
	HPV58 reverse	5' TCG CTG CAA AGT CTT TTT 3'
**HPV58**	HPV58 probe	5' FAM-CAT TCA ACG CAT TTC AAT TCG ATT T-BHQ1 3'
	EMC7 65 forward	5' CTT TCC CCA TGT TGC TTT AT 3'

	EMC7 65 reverse	5' CTG ACA ACC TCT GAT GTT TT 3'
	EMC7 65 probe	5' HEX-CAG AGC AAG ATA TGT GAA TTA CAT CAA-BHQ1 3'
	PBC Forward	5' ATC TGC AAA TGA ACA GYC TGA GA 3'
	PBC Reverse	5' CTT ACC TGA GGA GAC GGT GAC 3'
**White blood cell control**	PBC Probe	5' FAM-CYG AGG ACA CRG CTG TGT ATT ACT GTG C-BHQ1 3'
**CPP1 Spike-in control**	CPP1 Forward	5' CCA TGG ATG TAT TCG CCA GTT AC 3'
	CPP1 Reverse	5' TAA ATA TTG TGC TTC ACC TAC TCT AGT G 3'
	CPP1 Probe	5' HEX-TTG GCG TAG TTC TCC CGC TTA CCC CG-BHQ1 3'
	EMC7 250 Forward	5' AAG TAC TAC TGA GTA TGA TGT T 3'
	EMC7 250 Reverse	5' CTA GAT TTG CCA GAT GAT TTT 3'
**EMC7 250 / 65**	EMC7 250 Probe	5' FAM-AGT TGC CTG ATG TTT CTG AGT TCA T-BHQ1 3'
**Fragmentation control**	EMC7 65 forward	5' CTT TCC CCA TGT TGC TTT AT 3'
	EMC7 65 reverse	5' CTG ACA ACC TCT GAT GTT TT 3'
	EMC7 65 probe	5' HEX-CAG AGC AAG ATA TGT GAA TTA CAT CAA-BHQ1 3'

Primers and probes sequences used in the eight multiplex ddPCR. The PCR was standard Bio-Rad conditions (denaturation for 15 minutes followed by 40 cycles of 55°C for 60 seconds and 95°C for 15 seconds). The Pre-amplification mix contained the 6 HPV subtypes forward and reverse primers as well as the EMC7-65 forward and reverse primers.   .

Table S2. Samples used in the development of the multiplex HPV assays.

	**Non-amplified (alleles per uL ddPCR)**			**Pre-amplified (alleles per uL ddPCR)**			
	**HPV**	**EMC7-65**	**Ratio**	**HPV**	**EMC7-65**	**Ratio**	
HPV16	44.9	92.7	0.48	1970	5460	0.36	*
HPV16	35.3	56.4	0.63	917	2021	0.45	* †
HPV16	48.1	71.9	0.67	1801	4110	0.44	*
HPV16	30	13.7	2.19	3410	1885	1.81	
HPV18	8.7	26.2	0.33	781	2920	0.27	
HPV18	2.6	22.7	0.11	353	3160	0.11	
HPV31	0.28	32.6	0.01	20.6	4020	0.01	
HPV31	0.47	34.5	0.01	89.9	4590	0.02	
HPV31	1.1	59.7	0.02	82	6010	0.01	
HPV31	0.68	23.8	0.03	64.3	2800	0.02	
HPV31	0.48	34	0.01	63.7	3860	0.02	
HPV33	3.8	6.4	0.59	412	791	0.52	
HPV51	0.78	32.4	0.02	78.1	4130	0.02	
HPV51	10.4	34.4	0.30	1064	3480	0.31	
HPV58	45	87.3	0.52	5780	10900	0.53	

HPV positive samples from the departments cervix screening program was used in the development of the multiplex HPV assays and testing and validation of primers and probe and to develop positive control material, as well as to determine the optimal number of pre-amplification cycles and subsequent dilutions. The pre-amplification efficiency of the different HPV and EMC7-65 primer sets were evaluated by comparing the non-amplified and pre-amplified HPV / EMC7-65 ratios on positive plasma samples where residual material was available. The non- and pre- amplification ratios were highly conserved, especially when considering the stochastic variation of low copy numbers (alleles) in the non-amplified ddPCR reactions. * Sample subjected to one pre-amplification cycle less. † Sample subjected to an additional 2-fold dilution after pre-amplification.

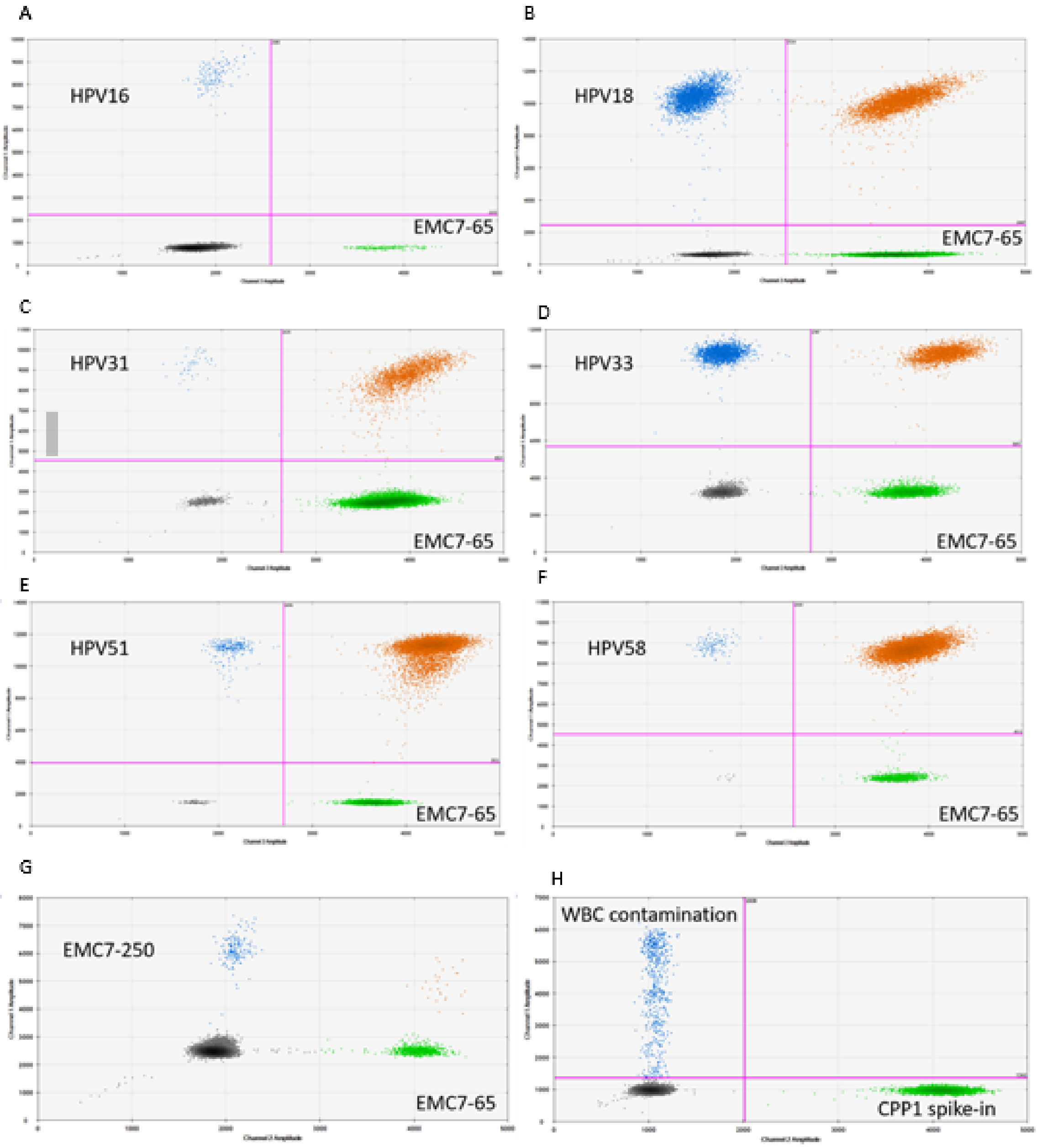

Figure 4. A-F: Examples of ddPCR positive samples for each HPV subtypes 16, 18, 31, 33, 51 and 58 (blue) as well as the EMC7-65 (green) after pre-amplification. **G**: Fragmentation control of plasma DNA using a 250- and a 65 base pair fragment of the EMC7 gene (ratio typical 0.2-0.5 in non-amplified cfDNA). **H**: Positive control for white blood cell (WBC) contamination and CPP1 spike-in (DNA purified from blood with added CPP1 DNA fragment). Orange dots are droplet containing DNA from both PCR targets. .
